# Site-Specific Immunomodulator: A Novel Treatment for Crohn's Disease

**DOI:** 10.1155/2015/231243

**Published:** 2015-05-12

**Authors:** Brian Bressler, Kevin P. Bethel, Ralf Kleef, Sophie L. Reynolds, Simon Sutcliffe, David W. Mullins, Hal Gunn

**Affiliations:** ^1^Gastrointestinal Research Institute (GIRI), Vancouver, BC, Canada V6Z 2K5; ^2^Freeport Family Wellness Center, P.O. Box F41325, Freeport, Bahamas; ^3^Institute for Immunotherapy and Integrative Oncology, 1130 Vienna, Austria; ^4^Qu Biologics Inc., Vancouver, BC, Canada V5T 4T5; ^5^Department of Microbiology and Immunology, Geisel School of Medicine at Dartmouth, Lebanon, NH 03756, USA

## Abstract

We investigated the mechanism of action, safety, and efficacy of the Site-Specific Immunomodulator (SSI) QBECO, a novel immunotherapy for Crohn's disease (CD). Using human monocytic THP-1 cells, we demonstrate that SSI QBECO (derived from the common colon bacteria *E. coli*) activates macrophages to an M1 phenotype (associated with enhanced capacity to eliminate bacteria and activate innate immune responses). We assessed SSI QBECO in a compassionate use protocol of ten adult patients with active CD. Patients with moderate to severe clinical symptoms receiving conventional CD treatments and/or complementary therapies were included, except patients receiving anti-TNF medications. SSI QBECO was self-administered subcutaneously every second day, for a minimum of 2.5 months and a maximum of 11 months. All 10 patients reported improvement of symptoms while on the SSI QBECO treatment. Seven patients reported full resolution of clinical symptoms during a course of SSI QBECO of at least three months. Three patients have experienced ongoing sustained clinical remission after discontinuing all medications, including SSI treatment. The longest case of clinical remission is still ongoing (>4 years). No serious severe adverse clinical events were reported. Collectively, we conclude that treatment with the immunoactive SSI QBECO was well tolerated and effective for treatment of Crohn's disease in this case series.

## 1. Introduction

Crohn's disease (CD) is a relapsing gastrointestinal inflammatory disorder [1] that results in 40–55% of patients requiring disease-related abdominal surgery within 10 years of diagnosis and a postoperative relapse rate of 44–55% after 10 years [2]. CD patients are also at increased risk for small bowel and colorectal cancers [3]. Its incidence is increasing amongst most ethnic groups [1, 4]. No curative treatment currently exists, and the effects of the long-term use of immunosuppressants and other medications used to treat its symptoms are poorly understood [2]. Moreover, because few, if any, experimental models of CD accurately resemble the human illness [1, 5, 6], the development of effective bench-to-bedside treatments is “increasingly difficult” [1].

Site-Specific Immunomodulators (SSIs) are a new and novel platform of immunotherapies created by Qu Biologics (QB) that may transcend this bench-to-bedside experimental impasse. SSIs are complex biologics designed to activate an innate immune response in a targeted, organ-specific manner and are each derived from a single species of inactivated bacteria that is a common cause of acute infection in the targeted organ. SSIs reverse the innate immune dysfunction and chronic inflammation that underlies both cancer and immune-mediated diseases, including CD, by stimulating the recruitment of a new wave of activated innate immune cells and the removal of the chronic source of unproductive inflammation.

The aims of this study were to (1) establish a conceptual framework for SSI mechanism of action using the gut-targeting drug SSI QBECO and (2) summarize the clinical and safety data for SSI QBECO in a case series from the compassionate-use program in patients with active CD. It has been suggested previously [5, 7–9] that impaired macrophage functionality plays a key role in the propagation of inflammation in CD by inhibiting the normal removal of damaged or infected cells and bacteria from the gut mucosa. Our central hypothesis was that by restoring normal macrophage functionality within the gut microenvironment of active CD patients through the administration of the SSI QBECO, we would effectively treat CD. These data form the precedent for a Phase 1/2 randomized, placebo-controlled, double-blind clinical trial that is currently evaluating the efficacy and safety of SSI QBECO in patients with moderate-to-severe CD.

## 2. Materials and Methods

### 2.1. SSI QBECO Drug Product Preparation

SSI QBECO, derived from an inactivated enteropathic strain of* E. coli*, was suspended in physiological saline containing 0.4% phenol as preservative. SSI QBECO was designed to target activated macrophage recruitment to the gastrointestinal tract, where enteropathic* E. coli* typically causes infection.

### 2.2. Human Monocytic Cell Line THP-1 Cell Culture

THP-1 cells were maintained in RPMI with L-glutamine (Fisher) supplemented with 50 *μ*M 2ME (Sigma Aldrich) and 10% FBS (Fisher). When ready to culture, cells were seeded at 2 × 10^6^/mL in 96- or 24-well tissue culture plates. PMA (200 ng/mL, Sigma-Aldrich) was added overnight to trigger differentiation to macrophages. After 20 hours (hrs), PMA media were removed and cells were washed twice in supplemented media.

### 2.3. Cytokine Dose Response Analysis

THP-1 cells were cultured with either LPS (used as a positive control) from* E. coli* (serotype 0111:B4 Sigma Aldrich, 0.01−10 *μ*g/mL) or with SSI QBECO (5 OD at 1 : 20, 1 : 200, 1 : 500, or 1 : 2500 dilutions) for 18 hrs, after which supernatants were removed and frozen for later analysis. Cytokine levels were quantified by specific ELISA for TNF-*α* and IL-1*β* (R&D Systems DuoSets). The limits of detection were 15.6 pg/mL and 3.91 pg/mL, respectively.

### 2.4. THP-1 Polarization

THP-1 cells were cultured in the presence of media for M0, IFN*γ* (20 ng/mL, Peprotech) and LPS (100 ng/mL) for M1, or IL-4 and IL-13 (both at 20 ng/mL, Peprotech) for M2 or with SSI QBECO drug product (1 : 20 or 1 : 500). After 18 hrs, RNA was extracted from cell pellets of polarized THP-1 cells using a PureLink RNA Mini Kit according to manufacturer's instructions (Ambion, Life Technologies). Genomic DNA was removed and cDNA was synthesized from 100 ng RNA using QuantiTect Reverse Transcription Kit (Qiagen). Real time PCR was performed with Taqman Fast Advanced Master Mix (Applied Biosystems) in the presence of 6-carboxyfluorescein- (FAM-) labeled primers for M1 genes (CCL19, CXCL11, CCR7, and TNF) and M2 genes (CCL13, CCL18, and FGL) (Applied Biosystems) using a StepOnePlus instrument (Applied Biosystems). Amplification conditions were 95°C for 20 seconds (s), 95° for 1 s before 60°C for 20 s for 40 cycles. Expression of M1 and M2 genes was normalized to 18SRNA (Applied Biosystems) for quantification. The housekeeping gene CT value was subtracted from the gene of interest CT (ΔCT). The difference was then calculated between the control (M2) and the other samples (ΔΔCT). The fold change was then calculated from this number (2^(−ΔΔCT)^).

### 2.5. Patients

Ten CD subjects between 24 and 44 years of age with moderate to severe clinical symptoms of active CD refractory to current treatments received the SSI QBECO according to a compassionate-use protocol (Table 1). Subjects receiving conventional CD treatments and/or complementary therapies were included; subjects receiving anti-TNF medications were excluded.

### 2.6. Informed Consent

Between July 2010 and January 2013, patients who had failed standard treatments for CD were, at the discretion of the treating physician, eligible for the compassionate-use of experimental treatments in Austria and The Bahamas. Signed patient informed consent was obtained from all patients prior to initiation of SSI QBECO treatment. Treatment of patients, according to a submitted protocol for SSI QBECO use, was approved by Bahamas Clinical Services for the eight patients treated in the Bahamas. The two CD patients enrolled in Austria were treated under the Austrian Named Patient Use Program (http://www.basg.gv.at/fileadmin/user_upload/L_I217_Compassionate_use_AT_en.pdf), consistent with prior reports [11]. The Bahamian and Austrian compassionate use programs were conducted in accordance with the 1975 Helsinki Declaration, as revised in 2008.

### 2.7. Treatment

Patients were taught to self-administer SSI QBECO treatment by subcutaneous injection every second day. Beginning with a dose of 0.05 mL, the dose was gradually increased (by 0.02 mL) until a 2.5–5.0 cm light pink local skin immune response (LSIR) was obtained as measured on the day following the injection, indicating the achievement of an adequate LSIR. Once this LSIR was achieved (or the maximum dose of 0.2 mL was reached), this customized dose was continued every second day until the end of the treatment course (minimum of 2.5 months to a maximum of 11 months). Dose, dose frequency, and side-effects were captured daily in a subject diary, and subjects were followed up closely by a physician (author Kevin P. Bethel or Ralf Kleef) who regularly assessed their adverse events and clinical symptoms of CD. Response and remission were assessed by the treating physician and were determined based on the physician's overall assessment of each patient's CD during the course of therapy. Assessments were made by office visit or by telephone consultation.

## 3. Results

### 3.1. Preclinical: SSI QBECO Induced M1-Skewed Responses in the THP-1 Monocyte Cell Line

**Figure 1:**
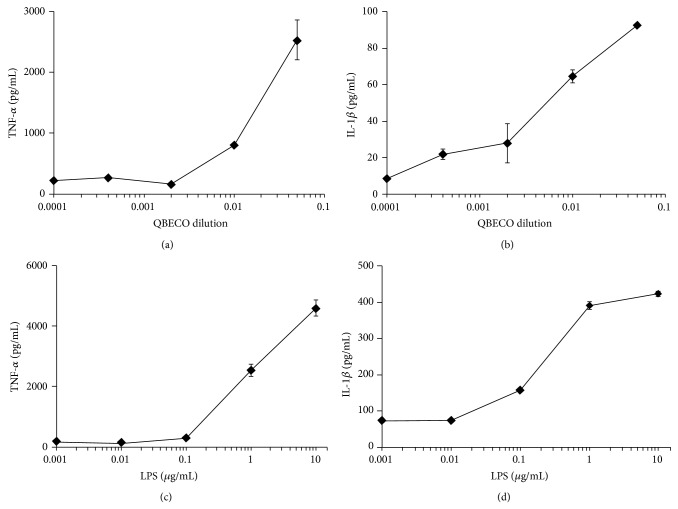
SSI QBECO- and LPS-induced cytokine release in THP-1 cells. THP-1 cells were activated overnight with PMA, then stimulated with diluted SSI QBECO (1 : 2500–1 : 20 dilution, (a)-(b)) or LPS (0.01–10 *μ*g/mL) for 18 hrs. Supernatants were removed and TNF-*α* ((a), (c)) or IL-1*β* ((b), (d)) was assessed by specific ELISA. Data are means ± standard deviation of triplicate assessments from one of three similar experiments.

**Figure 2:**
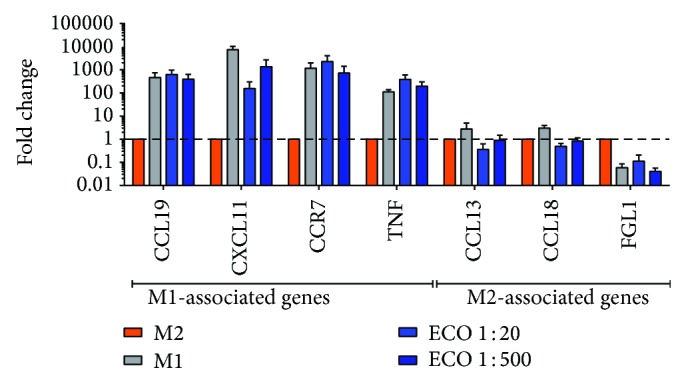
M1 and M2 mRNA expression in polarized THP-1 cells. THP-1 cells were cultured for 18 hrs with IFN*γ* and LPS (generating an M1 phenotype), IL-4 and IL-13 (generating an M2 phenotype), or SSI QBECO (1 : 20 or 1 : 500 dilution). Specific gene expression of M1- (CCL19, CXCL11, CCR7, and TNF-*α*) and M2- (CCL13, CCL18, and FGL1) associated genes was quantified by qPCR. Data are fold change in gene expression, relative to M2 polarized cells.

We assessed the capacity for SSI QBECO to induce macrophage cytokine and gene expression* in vitro*, using the human monocytic cell line THP-1. SSI QBECO treatment of THP-1 cells, following activation to macrophage phenotype by overnight culture with PMA, induced the production of proinflammatory, M1-associated cytokines (IL-1*β* and TNF-*α*, Figure 1) in a dose-dependent manner that resembles LPS-activated cytokine production. Further, SSI QBECO induced the expression of a panel of M1-associated, but not M2-associated [12], gene products (Figure 2). These data demonstrate that SSI QBECO preferentially induces the M1-type differentiation of macrophages. This observation provides, in part, a rationale for the evaluation of SSI QBECO in patients with chronic active CD.

### 3.2. Clinical: Compassionate-Use Clinical Program Patient Response

The patient demographics are described in Table 1. Patient 5 had a history of a rectovaginal fistula; at the time of receiving SSI therapy, she had no symptoms related to this pathology. Patient 5 received SSI therapy for treatment of her luminal symptoms. Patient 5 did not respond to 5ASA, and no other medical therapies were tried prior to SSI treatment.

Ten of 10 patients reported improvement of symptoms while on SSI QBECO treatment. Seven patients reported full resolution of clinical symptoms during a course of SSI QBECO treatment of at least three months. Three patients have experienced ongoing sustained clinical remission after discontinuing all medications, including SSI treatment. The longest case of clinical remission reported is still ongoing, after more than four years. All three patients have had a follow-up colonoscopy or CT scan with confirmation of remission of CD (Table 2).

### 3.3. Adverse Events

With a maximum follow-up period of 52 months, no treatment-related serious adverse events have been observed or reported to date. The only reported treatment-related adverse events were 2-3 episodes of transient fever lasting 12–24 hrs in three patients (which resolved without treatment within 12–24 hrs) and a larger than anticipated transient local skin immune response to initial treatment dose in one patient, which was corrected with appropriate dose reduction.

## 4. Discussion

**Figure 3:**
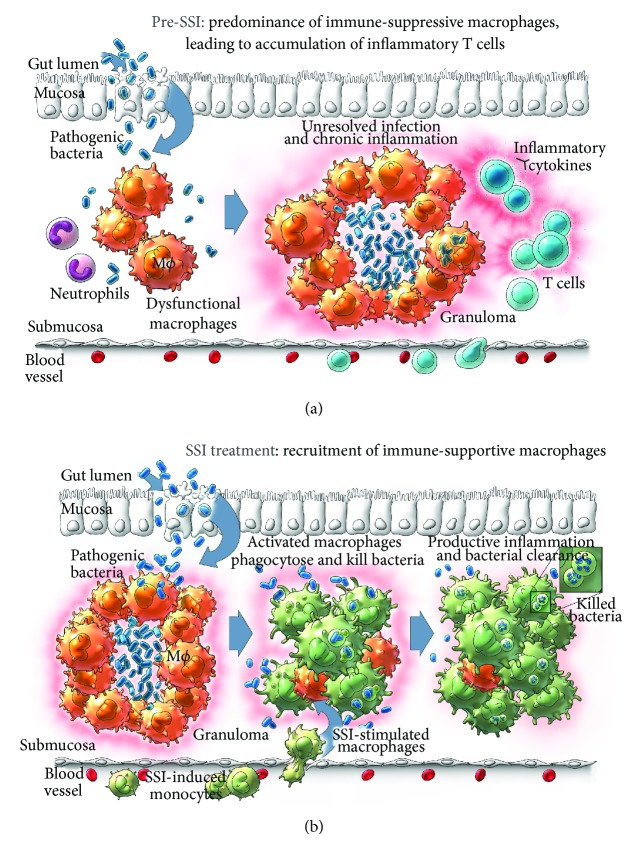
Proposed model of SSI-mediated M1 macrophage accumulation and effector function in CD. (a) Pre-SSI microenvironment of CD, with predominance of dysfunctional macrophages and unresolved infection/chronic inflammation from lack of initial acute inflammatory response. (b) Post-SSI microenvironment, with recruited immune-supportive M1 macrophages capable of clearing the bacteria that initiate acute inflammatory responses and pave the way for M2 macrophages to engage in healing and rebuilding activities.

While CD is an immune-mediated disease, it is not an autoimmune disease [7, 13]. The CD* immunodeficiency hypothesis *(IDH), dating in part to the late 1970s [14], asserts that (1) infectious agents breach the bowel mucosal lining, (2) a predisposing innate immune deficiency prompts an ineffective acute physiological inflammation and impaired recruitment of phagocytes, (3) the neutrophil-based phagocytes become overwhelmed, (4) macrophages therefore attempt to contain the pathogens but are less capable at this function, and (5) these macrophages drive a chronic but inefficient immune response [5] (Figure 3(a)). This hypothesis was perceived to be in conflict with the apparent clinical efficacy of immunosuppressive medications used to treat CD symptoms and its popularity waned. However, new supporting IDH evidence [5, 6, 9, 15, 16] suggests that there are, in fact, several discreet phases of immunodeficient responses in CD: the first involving poor cytokine production by resident tissue macrophages followed by diminished neutrophil recruitment and subsequent chronic inflammation [16]. Indeed, it is this compensatory chronic inflammation response that is suppressed by medications such as azathioprine, infliximab, and adalimumab (which have been the focus of current CD treatment).

By extension, treatment of the deficient first acute innate immune response could also address the subsequent inefficient inflammation phase that results in clinical manifestation of CD and thereby potentially reduce or eliminate the need for the long-term use of immunosuppressive medications in CD patients. SSIs initiate such a response, one that is innate, acute, functional, organ-specific, and nontoxic to the tissue in which the respective bacterial species commonly causes infection (Figure 3(b)). In preclinical models, the SSI QBECO specifically targets the gut and we have demonstrated that it stimulates innate immune responses in the colon and gastrointestinal tract (unpublished observation). Since macrophage defect or deficiency, in particular, may be the underlying trigger for CD, the SSI QBECO was designed to induce organ-specific macrophage recruitment and activation, resulting in the clearance of bacterial infection and necrotic debris.

The deficiency observed in the first innate mucosal immune response in CD patients may be that of defective M1 macrophages, and the resultant inflammation phase of clinical symptoms may be caused by the accumulation of other dysfunctional (potentially M2) macrophages concurrently with the unresolved infection. While M2 macrophages lead to tissue regeneration and repair [17], they are incapable of either killing/removing bacteria or instigating an effective innate immune response, which is the hallmark of M1 macrophages. This ultimately Sisyphean effort leads to cyclic, but futile, attempts at repair with the simultaneous ineffective clearance of the initial infection. Thus, we hypothesized that the SSI QBECO, which drives M1 macrophage polarization* in vitro*, may also drive M1 expansion in patients and thereby lead to more effective treatment of the underlying cause of CD, and not just its symptoms. By extension, once the underlying source of inflammation was cleared, the symptoms characteristic of CD would resolve in patients who received the SSI QBECO medication. We observed clinical remission during administration of the SSI QBECO in a compassionate-use context in seven of 10 patients who had moderate to severe clinical symptoms of active CD at baseline, with the remaining three of 10 noting improvement of their symptoms during treatment. Three of the 10 patients have experienced ongoing long-term remission. Based on these initial findings, and the recognized limitations of compassionate-use studies regarding causal attribution and mechanism of action, induction of clinical response and remission by SSI QBECO therapy is now being evaluated through a Phase 1/2 randomized, placebo-controlled, double-blind clinical trial. QB is currently characterizing the unique qualities of SSI-stimulated M1 macrophage populations, and, in future studies, QB will explore the potentially unique genetic differences of the long-term responder cohort in order to develop assays that would identify,* a priori*, likely future candidates for which treatment would be efficacious and warranted.

## Figures and Tables

**Table 1:** Characteristics of patients with CD.

					Montreal Classification									
Patient	Sex	Date of birth	Age at diagnosis of CD (yrs)	Disease prior to SSI (yrs)	Age at diagnosis of CD	Location of CD	Behavior	Previous resection	Prior CD therapy	Past use of TNF-*α* blocker	Medication refractoriness prior to SSI	Symptoms at initiation of SSI	Symptom severity at initiation of SSI	Medications at initiation of SSI
1	M	11-May-83	23	4	A2	L3	B1	0	Prednisone, Imuran	No	Steroid dependant, Imuran	Pain, diarrhea, and weight loss	Severe	Imuran, Prednisone

2	F	07-Jan-85	24	2	A2	L1	B3	0	Prednisone, antibiotics	No	Steroid dependant	Pain, diarrhea, RLQ abscess, and weight loss	Moderate	Prednisone

3	F	01-Dec-77	4	30	A1	L2	B2	1	Prednisone, Remicade	Yes	Steroid dependant, Remicade	Pain, diarrhea, and anemia	Severe	Prednisone

4	F	29-Jan-88	21	3	A2	L3	B3p	1	Prednisone, Imuran, Remicade	Yes	Imuran	Pain, diarrhea, and fistulae	Severe	Imuran

5	F	05-Sep-73	35	3	A2	L3	B3p (RV fistula)	0	5ASA	No	5ASA	Pain, diarrhea	Moderate	5ASA

6	F	13-Sep-78	20	13	A2	L2	B1	0	Prednisone, Purinethol, 5ASA, Entocort	No	Purinethol, 5ASA, Entocort	Pain, diarrhea	Moderate	none

7	F	22-Sep-73	24	15	A2	L2	B1	0	Prednisone, Remicade, 5ASA	Yes	Remicade, 5ASA	Pain, diarrhea	Moderate	5ASA

8	M	04-Sep-85	24	3	A2	L2	B1	0	Prednisone, 5ASA	No	5ASA	Pain, diarrhea, and weight loss	Severe	none

9	M	01-Oct-76	19	16	A2	L3 + L4	B3p	1	Prednisone, Remicade, 5ASA	Yes	Steroid dependant, Remicade, 5ASA	Pain, diarrhea, and fistulae	Severe	Prednisone

10	F	07-Nov-67	45	0.5	A3	L3	B3p	0	Prednisone, Imuran, 5ASA	Yes	Steroid dependant, Imuran, 5ASA	Pain, diarrhea	Moderate	Prednisone, 5ASA

Patient demographic criteria were based on the Montreal Classification of CD [10]: Age at diagnosis of CD: A1: ≤16 years, A2: 17–40 years, A3: >40 years; Location of Crohn's disease: L1: terminal ileum, L2: colon, L3: ileocolon, L4: upper GI, L1 + L4: terminal ileum + upper GI, L2 + L4: colon + upper GI, and L3 + L4; ileocolon + upper GI; and Behavior: B1: nonstricturing, nonpenetrating, B2: stricturing, B3: penetrating, B1p: nonstricturing, nonpenetrating + perianal, B2p: stricturing + perianal, and B3p: penetrating + perianal.

**Table 2:** Patient response to SSI QBECO treatment.

Patient	QBECO SSI therapy duration	Response while on SSI treatment	Status after SSI treatment	Current medications	Confirmation of ongoing remission
1	11 months	Remission	Ongoing remission 3.8 years	None	Colonoscopy

2	3 months	Remission	Ongoing remission 2.8 years	None	CT scan

3	6 months	Remission	Remission 1.4 years, disease recurrence	Standard care	n/a

4	8 months	Remission, including perianal fistulae	Ongoing remission 2.2 years	None	Colonoscopy

5	3.5 months	Remission	Remission 1.2 years, disease recurrence	Standard care	n/a

6	5 months	Remission	Remission 3 months, disease recurrence	Standard care	n/a

7	7.5 months	Remission	Recurrence post-SSI Less severe symptoms	Standard care	n/a

8	6.5 months	Improvement	Symptom return post-SSI Less severe symptoms	Standard care	n/a

9	5 months	Improvement, reduction in prednisone	Symptom return post-SSI	Standard care	n/a

10	2.5 months	Improvement	Symptom return post-SSI	Standard care	n/a

Standard care: patient is being treated by other approved CD drugs.
